# Detection of soluble interleukin-2 receptor and soluble intercellular adhesion molecule-1 in the effusion of otitis media with effusion

**DOI:** 10.1155/S0962935195000561

**Published:** 1995-09

**Authors:** T. Ganbo, K.-I. Hisamatsu, H. Inoue, K. Kikushima, A. Mizukoshi, Y. Murakami

**Affiliations:** 1 Department of Otolaryngology Yamanashi Medical University Yamanashi Japan; 2 Department of Plastic & Reconstructive Surgery St Marianna University School of Medicine Kawasaki Japan

## Abstract

We measured sIL-2R, TNF-α and sICAM-1 in the sera and middle ear effusions (MEEs) of patients with otitis media with effusion (OME). Although there was no signmcant difference between the sIL-2R levels of the serous and mucoid MEEs, they were significantly higher than serum sIL-2R levels of OME patients and healthy controls. TNF-α levels of the mucoid MEEs were significantly higher than those of the serous type. However, TNF-α was rarely detected in the sera of OME patients or healthy controls. We observed significant differences between the serous and mucoid MEEs with respect to their sICAM-1 levels, which were also higher than serum slCAM-1 levels of OME patients and healthy controls. Our findings suggested that IL-2, TNF-α and ICAM-1 could be significantly involved in the pathogenesis of OME through the cytokine network.


Mediators of Inflammation 4, 350–354 (1995)

### Detection of soluble interleukin-2 receptor and soluble intercellular adhesion molecule-1 in the effusion of otitis media with effusion

T. Ganbo,[1,CA] K.-I. Hisamatsu,[1] H. Inoue,[2] K. Kikushima,[1] A. Mizukoshi[1] and Y. Murakami[1]

[1]Department of Otolaryngology, Yamanashi Medical University, Yamanashi, Japan; [2]Department of Plastic & Reconstructive Surgery, St Marianna University School of Medicine, Kawasaki, Japan

[CA]Corresponding Author

**We measured sIL-2R, TNF-α and sICAM-1 in the sera and middle ear effusions (MEEs) of patients with otitis media with effusion (OME). Although there was no significant difference between the sIL-2R levels of the serous and mucoid MEEs, they were significantly higher than serum sIL-2R levels of OME patients and healthy controls. TNF-α levels of the mucoid MEEs were significantly higher than those of the serous type. However, TNF-α was rarely detected in the sera of OME patients or healthy controls. We observed significant differences between the serous and mucoid MEEs with respect to their sICAM-1 levels, which were also higher than serum sICAM-1 levels of OME patients and healthy controls. Our findings suggested that IL-2, TNF-α and ICAM-1 could be significantly involved in the pathogenesis of OME through the cytokine network.**

**Key words:** Otitis media with effusion, Soluble IL-2R, Soluble ICAM-1, TNF-α.

### Introduction

Interleukin-2 (IL-2) is one of the most well characterized cytokines. It stimulates the proliferation of activated T-lymphocytes,[1,2] and binds to the IL-2 receptor (IL-2R) which is composed of three distinct subunits, namely α, β and γ chains.[3–5] In patients with rheumatoid arthritis,[6] adult T cell leukaemia,[7] Kawasaki disease[8] and bronchial asthma,[9] the serum levels of soluble IL-2R (sIL-2R), which is the released extracellular domain of the α-chain of IL-2R, are elevated. Moreover, activated T-lymphocytes are reported not only to elaborate interferon-gamma (INF-γ)[10] and to cause macrophages to produce tumour necrosis factor-alpha (TNF-α),[11] but also to regulate the expression of intercellular adhesion molecule-1 (ICAM-1) on the endothelium indirectly through such media as TNF-α and INF-γ, resulting in the recruitment of inflammatory cells.[12] ICAM-1 belongs to the immunoglobulin supergene family[13] and acts a ligand for leukocyte function-associated antigen-1 (LFA-1)[14] and integrin corresponding to the $\beta_2$ group (Mac-1).[15] It plays important roles in intercellular interaction when leukocytes adhere and transmigrate to a focus. Thus, soluble ICAM-1 (sICAM-1), a soluble form of ICAM-1, is detected in the focus because of the shedding of ICAM-1 as well as the expression of ICAM-1 on the cell surface induced by TNF-α and INF-γ.[16,17]

In otitis media with effusion (OME), predominant neutrophils with lymphocytes and macrophages are seen in the effusion and the submucosa of the middle ears.[18,19] Cytokines form a network together with inflammatory cells and adhesion molecules, and play important roles in the inflammatory disorders. It is necessary to investigate the effects of cytokines and adhesion molecules on the pathogenesis of OME. However, there have been few reports about sIL-2R and sICAM-1 in cases with OME. Therefore, we studied sIL-2R, TNF-α and sICAM-1 in the middle ear effusion and the sera of patients with OME.

### Materials and Methods

*Subjects:* We collected middle ear effusions (MEEs) using Juhn Tym-Taps (Xomed-Treace, Jacksonville, FL, USA) from 31 patients with OME (17 males and 14 females, ranging in age from 3 to 79 years with a mean of 22.9 years) when they had undergone myringotomy. The MEEs were classified into two groups, the serous and the mucoid types. While 16 patients (nine males and seven females with a mean age of 22.9 years, ranging from 4 to 76 years) belonged to the serous type, 15 others (eight males and seven females with a mean age of 23.0 years, ranging from 3 to 79 years) belonged to the mucoid type. Serum samples were also obtained from all 31 patients with OME, and from ten healthy volunteers who served as controls. MEEs and sera were collected with the informed consent of the patients and/or their families.

© 1995 Rapid Science Publishers

*Preparation of MEEs and sera:* One to 3 volumes of dithiothreitol[20] (Sputolysin®; Behring Diagnostics, La Jolla, CA, USA) were added to the MEEs and mixed with a vortex mixer to dissolve in phosphate-buffered saline (PBS). Finally, the sample was diluted to the concentration of 2 to 10 volumes of the MEE with dithiothreitol and PBS. These samples were centrifuged at 1500 × **g** for 10 min, and the supernatants were collected and stored at −80°C until they were assayed. The sera were also stored in the same way.

*The assays for sIL-2R, TNF-α and sICAM-1:* The concentrations of sIL-2R, TNF-α and sICAM-1 were measured using commercially available enzyme-linked immunosorbent assays (ELISA). The assays of sIL-2R, TNF-α and sICAM-1 were performed in duplicate with cell-free interleukin-2 receptor bead assay kit (T cell Diagnostic Co., MA, USA), human TNF-α immunoassay kit (Research and Diagnostics Systems, MN, USA) and human ICAM-1 ELISA kit (Serotec, Oxford, England), respectively. The concentrations of sIL-2R, TNF-α and sICAM-1 in the test samples were determined from the calibration curve.

For the measurement of sIL-2R, TNF-α and sICAM-1 in the MEE, the effect of the MEE itself on these assays was preliminarily tested but no significant effect was found. Briefly, three kinds of stock solutions obtained from surplus MEEs were prepared for the test. An equal volume of each of standard sIL-2R, TNF-α and sICAM-1 was added to each stock solution and the actual increase in sIL-2R, TNF-α and sICAM-1 concentrations was compared with the calculated value.

*Statistical analysis:* The statistical significance of the difference between the recorded values was determined at $p < 0.01$ by Student's *t*-test.

### Results

The levels of sIL-2R in the MEEs and sera are illustrated in Fig. 1. The mean level of sIL-2R in the mucoid MEEs was 15000 U/ml, while the mean level in the serous MEEs was 9500 U/ml. There was no significant difference between the sIL-2R levels of the two types of MEEs. However, the sIL-2R levels in both types were significantly higher than those of the sera of OME patients, which was 326 U/ml on the average. Moreover, there was no significant difference between the sera of OME patients and those of the healthy controls (310 U/ml).

The levels of TNF-α in the MEEs and sera are illustrated in Fig. 2. The mean level of TNF-α in the mucoid MEEs was 300 pg/ml, whereas the mean level in the serous MEEs was 66 pg/ml. There was a significant difference between the TNF-α levels of the two types of MEEs. TNF-α was detected in the sera of five out of 19 cases and their mean level was 7.8 pg/ml. The TNF-α levels of the two types of MEEs were significantly different from those of the sera of the detected cases. The mean of the total serum levels of TNF-α including 14 undetected cases was 2.1 pg/ml, which was as high as that of the healthy controls (2.1 pg/ml).

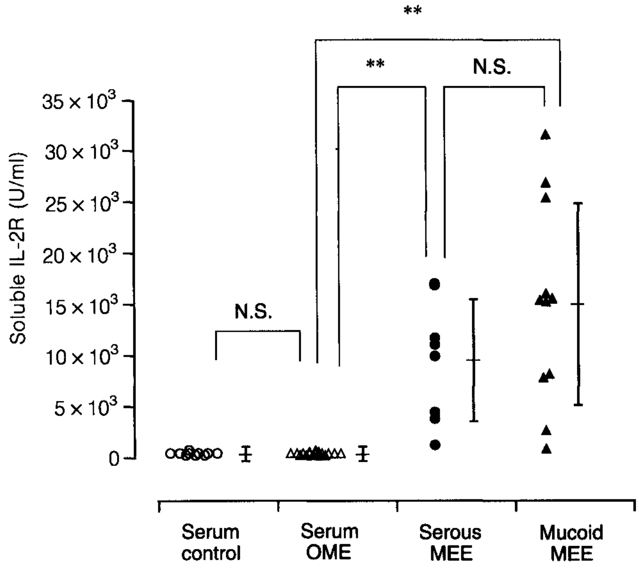

FIG. 1. The sIL-2R levels in the serous ($n = 8$) and mucoid MEEs ($n = 11$) of OME patients are shown along with serum levels of sIL-2R in healthy control volunteers ($n = 10$) and OME patients ($n = 19$). Each bar represents mean and S.D. $^{**}p < 0.01$.

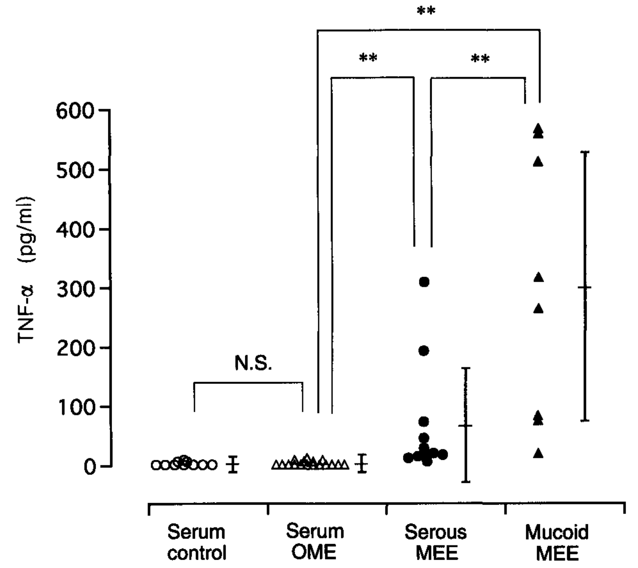

FIG. 2. The TNF-α levels in the serous ($n = 11$) and mucoid MEEs ($n = 8$) of OME patients are shown along with serum levels of TNF-α in healthy control volunteers ($n = 10$) and OME patients ($n = 19$). Each bar represents mean and S.D. $^{**}p < 0.01$.

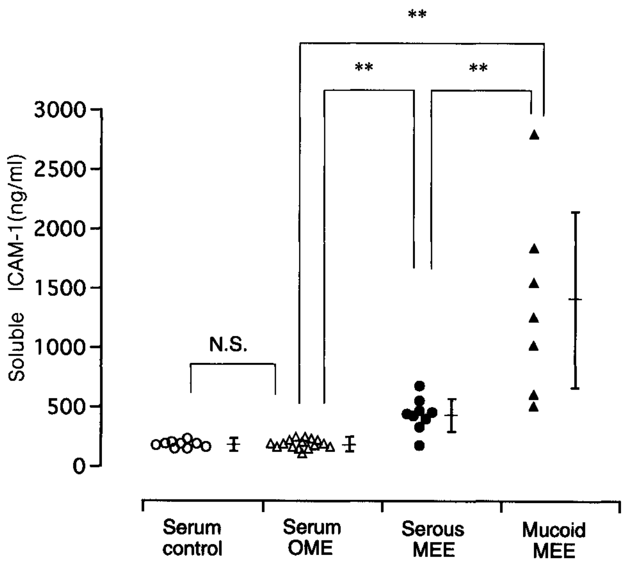

FIG. 3. The sICAM-1 levels in the serous ($n$=9) and mucoid MEEs ($n$=7) of OME patients are shown along with serum levels of sICAM-1 in healthy control volunteers ($n$=10) and OME patients ($n$=16). Each bar represents mean and S.D. $^{**}p < 0.01$.

The levels of sICAM-1 in the MEEs and sera are illustrated in Fig. 3. The mean level of sICAM-1 in the mucoid MEEs was 1440 ng/ml, whereas the mean level in the serous MEEs was 430 ng/ml. Thus, a significant difference was observed between sICAM-1 levels of the two types of MEEs. Furthermore, the levels of sICAM-1 in the two types of MEEs were significantly different from those of the sera of the OME patients because the mean serum level was 180 ng/ml. However, there was no significant difference between the sera of the OME patients and those of the healthy controls (180 ng/ml).

### Discussion

The sIL-2R levels in the serous and mucoid types of MEEs were high, their mean values being 9500 U/ml and 15 000 U/ml, respectively. These values were extremely high as compared with the sIL-2R levels of bronchoalveolar lavage fluid (BALF) of asthma (42 U/ml) and miliary tuberculosis (1320 U/ml),[9] nasal secretion of nasal allergy (1510 U/ml)[21] and synovial effusion of rheumatoid arthritis (1170 U/ml).[6] Even in the severe cases of acute asthma,[22] extensive pulmonary tuberculosis[23] and acute-phase Kawasaki disease,[8] it was reported that the mean values of serum sIL-2R levels were about 570 U/ml, 2700 U/ml and 3700 U/ml, respectively. However, sIL-2R level of acute adult T cell leukaemia which was beyond 10 000 U/ml in serum,[7] was as high as that of MEE. In our study, the mean level of sIL-2R in the sera of OME patients was 326 U/ml, which was the same as that of the healthy controls (310 U/ml). These data suggest that the expression of IL-2R on activated T lymphocytes and the binding of IL-2 to its receptor might be concerned with local pathogenesis of OME in the middle ear. Yellon *et al.*[24] also reported that IL-2 was detected in the MEEs of 54% of OME patients. However, there is a possibility that IL-2 might be biologically a little unstable or technically difficult to detect as compared with sIL-2R, because sIL-2R was detectable in all our cases.

There are a few reports which claim that TNF-α was detected in the MEEs of OME,[24–26] although its values were expressed with the unit of pg/mg of total protein. Particularly, the mucoid type effusion is too viscous to dissolve in PBS. In our methods, we used dithiothreitol and liquefied the mucoid pellet to dissolve it in PBS.[20] We expressed TNF-α levels in the effusion with the unit of pg/ml and could compare TNF-α values with those of other diseases. In the BALFs[27] and sputa[28] of patients with active asthma, mean TNF-α levels were 578 pg/ml and 1783 pg/ml, respectively. In children with leukaemia, the mean level of TNF-α in the sera was 63.6 pg/ml while it was 21.5 pg/ml in the solid tumours.[29] We found that the mean TNF-α level in the mucoid type effusions was similar to that in BALFs of asthma patients.

Chihara *et al.*[30] reported that sICAM-1 levels of sputa and sera of asthma patients were 8.6 ng/ml and 350 ng/ml, respectively, and that the sICAM-1 level in the normal human sera was 200 ng/ml. It means that the mean serum level of sICAM-1 in asthma was 1.75 times higher than that in the healthy control and that its sputum level could be disregarded as it was too low as compared with the serum level of control subjects. In our study, sICAM-1 levels in the serous and mucoid MEEs were 5.3 and 7.8 times higher than those in the control sera. sICAM-1 levels in various other diseases have also been reported. In patients with rheumatoid arthritis,[31] serum and synovial fluid levels of sICAM-1 were 1.2 and 1.5 times higher than the serum levels of the normal controls, respectively. Serum levels of sICAM-1 in patients with nasal allergy[32] and malignant diseases with metastasis[16] were 1.4 and 5.7 times higher than those of the healthy controls, respectively. These data indicate that sICAM-1 levels detected in the MEEs of OME were very high while the serum levels were the same as those of the healthy controls. Himi *et al.* also reported that sICAM-1 levels of MEEs were significantly higher than those of the healthy control sera.[33] However, they could not make any distinction between the various types of the MEEs because their sICAM-1 levels were expressed in ng/mg of total protein, which was different from our unit ng/ml. However, it is not surprising that sICAM-1, a soluble component of ICAM-1, was highly detectable in the MEEs. It is supposed that ICAM-1 might play an important role in inflammatory disorders of the middle ear. Our investigation suggested that ICAM-1 might cause a difference in the production and/or accumulation of MEE because there was a significant difference between the sICAM-1 levels of the serous and mucoid MEEs. This supposition was highly acceptable also for the fact that there was significant difference between the TNF-α levels of the two types of MEEs and that TNF-α also activates endothelium, causing the expression of adhesion molecules such as ICAM-1.[12]

The physiological significance of the shedding of sIL-2R and sICAM-1 remains unknown. However, we supposed that they might be playing some role in inflammatory disorders. There is a possibility that they could serve as useful markers of inflammation, and may also have inhibitory effects on the inflammatory cascade of reactions, because sIL-2R inhibits the functional response elicited by IL-2,[6] and sICAM-1 prevents cell adherence to native ICAM-1.[16–17] IL-2 induces the proliferation of activated lymphocytes and ICAM-1 also facilitates adhesion and transmigration of neutrophils.[34] These inflammatory cells invade the middle ear of OME patients. In cases of serous types of OME, it was reported that a larger number of neutrophils and lymphocytes were observed in the MEEs and that more severe inflammatory signs were shown in the submucosa, as compared with mucoid types.[18,19] However, particularity in our study, sICAM-1 levels of mucoid MEEs were significantly higher than those of serous MEEs. These morphological findings of cellular infiltration, which might be induced by ICAM-1, disagreed with sICAM-1 levels detected in the MEEs. Therefore, it is necessary to investigate the more detailed roles of sIL-2R and sICAM-1 in the pathogenesis of OME.

It has been reported that various chemical mediators such as leukotrienes, prostaglandins[35] and platelet activating factor[36] were detectable in the MEEs of OME. These chemical mediators are responsible at least partly for the pathogenesis of OME because they could induce hypersecretion and inhibit mucociliary transport systems in the middle ear, causing an accumulation of the effusion.[37,38] However, these mediators could also be secreted by inflammatory cells that infiltrated into the focus. Furthermore, these inflammatory cells are regulated by the cytokine network that consists of cytokines and related factors. Our investigation indicated the possibility that IL-2, TNF-α and ICAM-1 could affect the pathogenesis of OME through the inflammatory cells and the chemical mediators derived from these cells.

### References

1. Morgan DA, Ruscetti FW, Gallo R. Selective *in vitro* growth of T-lymphocytes from normal human bone marrows. *Science* 1976; **193:** 1007–1008.
2. Smith KA. T-cell growth factor. *Immunol Rev* 1980; **51:** 337–357.
3. Nikaido T, Shimizu A, Ishida N, *et al.* Molecular cloning of cDNA encoding human interleukin-2 receptor. *Nature* 1984; **311:** 631–635.
4. Hatakeyama M, Tsudo M, Minamoto S, *et al.* Interleukin-2 receptor beta chain gene: generation of three receptor forms by cloned human alpha and beta chain cDNA's. *Science* 1989; **244:** 551–556.
5. Takeshita T, Asao H, Suzuki J, Sugamura K. An associated molecule, p64, with high-affinity interleukin 2 receptor. *Int Immunol* 1990; **2:** 477–480.
6. Symons JA, Wood NC, Di Giovine FS, Duff GW. Soluble IL-2 receptor in rheumatoid arthritis. Correlation with disease activity, IL-1 and IL-2 inhibition. *J Immunol* 1988; **141:** 2612–2618.
7. Yasuda N, Lai PK, Stephen HI, *et al.* Soluble interleukin 2 receptors in sera of Japanese patients with adult T cell leukemia mark activity of disease. *Blood* 1990; **71:** 1021–1026.
8. Lang BA, Silverman ED, Laxer RM, Rose V, Nelson DL, Rubin LA. Serum-soluble interleukin-2 receptor levels in Kawasaki disease. *J Pediatr* 1990; **116:** 592–596.
9. Park CS, Lee SM, Uh ST, *et al.* Soluble interleukin-2 receptor and cellular profiles in bronchoalveolar lavage fluid from patients with bronchial asthma. *J Allergy Clin Immunol* 1993; **91:** 623–633.
10. Corrigan CJ, Kay AB. CD4-T lymphocyte activation in acute severe asthma. Relationship to disease severity and atopic status. *Am Rev Respir Dis* 1990; **141:** 970–977.
11. Gifford GE, Lohmann-Matthes ML. Gamma interferon priming of mouse and human macrophages for induction of tumor necrosis factor production by bacterial lipopolysaccharide. *J Natl Cancer Inst* 1987; **78:** 121–124.
12. Pober JS, Gimbrone Jr MA, Lapierre LA, Mendrick DL, Fiers W, Rothlein R, Springer TA. Overlapping patterns of activation of human endothelial cells by interleukin-1, tumor necrosis factor, and immune interferon. *J Immunol* 1986; **137:** 1893–1896.
13. Calderon E, Lockey RF. A possible role for adhesion molecules in asthma. *J Allergy Clin Immunol* 1992; **90:** 852–865.
14. Marlin SD, Springer TA. Purified intercellular adhesion molecule-1 (ICAM-1) is a ligand for lymphocyte function-associated antigen (LFA-1). *Cell* 1987; **51:** 813–819.
15. Smith CW, Marlin SD, Rothlein R, Toman C, Anderson DC. Cooperative interactions of LFA-1 and Mac-1 with intercellular adhesion molecule-1 in facilitating adherence and transendothelial migration of human neutrophils *in vitro*. *J Clin Invest* 1989; **83:** 2008–2017.
16. Tsujisaki M, Imai K, Hirata H, *et al.* Detection of circulating intercellular adhesion molecule-1 antigen in malignant diseases. *Clin Exp Immunol* 1991; **85:** 3–8.
17. Becker JC, Dummer R, Hartmann AA, Burg G, Schmidt RE. Shedding of ICAM-1 from human melanoma cell lines induced by IFN-γ and tumor necrosis factor-α; Functional consequences of cell-mediated cytotoxicity. *J Immunol* 1991; **147:** 4398–4401.
18. Sade J. Pathology and pathogenesis of serous otitis media. *Arch Otolaryngol* 1966; **84:** 297–305.
19. Ishii T, Toriyama M, Suzuki J. Histopathological study of otitis medea with effusion. *Ann Otol Rhinol Laryngol* 1980; **89(suppl 68):** 83–86.
20. Hirsch SR, Zastrow JE, Kory RC. Sputum liquefying agents: a comparative *in vitro* evaluation. *J Lab Clin Med* 1969; **74:** 346–353.
21. Hisamatsu K, Ganbo T, Nakazawa T, Horiguchi S, Shimomura S, Murakami Y. Elevation of soluble interleukin-2 receptor levels in nasal allergy. *Mediators of Inflammation* 1995; **4:** 39–42.
22. Brown PH, Crompton GK, Greening AP. Proinflammatory cytokines in acute asthma. *Lancet* 1991; **338:** 590–593.
23. Takahashi S, Setoguchi Y, Nukiwa T, Kira S. Soluble interleukin-2 receptor in sera of patients with pulmonary tuberculosis. *Chest* 1991; **99:** 310–314.
24. Yellon RF, Leonard G, Marucha PT, *et al.* Characterization of cytokines present in middle ear effusions. *Laryngoscope* 1991; **110:** 165–169.
25. Himi T, Suzuki T, Kodama H, Takezawa H, Kataura A. Immunologic characteristics of cytokines in otitis media with effusion. *Ann Otol Rhinol Laryngol* 1992; **101:** 21–25.
26. Juhn SK, Garvis WJ, Lees CJ, Le CT, Kim CS. Determining otitis media severity from middle ear fluid analysis. *Ann Otol Rhinol Laryngol* 1994; **103(Suppl 163):** 43–45.
27. Broide DH, Lotz M, Cuomo AJ, Coburn DA, Federman EC, Wasserman SI. Cytokines in symptomatic asthma airways. *J Allergy Clin Immunol* 1992; **89:** 958–967.

28. Taki F, Kondoh Y, Matsumoto K, Takagi K, Satake T. Tumor necrosis factor in sputa of patients with bronchial asthma on exacerbation. *Arerugi* 1991; **40:** 643–646.
29. Abrahamsson J, Carlsson B, Mellander L. Tumor necrosis factor-alpha in malignant disease. *Am J Pediatr Hemato Oncol* 1993; **15:** 3664–3669.
30. Chihara J, Yamamoto T, Kurachi D, Nakajima S. Soluble ICAM-1 in sputum of patients with bronchial asthma. Lancet 1994; **343:** 1108.
31. Koch AE, Shah MR, Harlow LA, Lovis RM, Pope RM. Soluble intercellular adhesion molecule-1 in arthritis. *Clin Immunol Immunopathol* 1994; **71(2):** 208–232.
32. Terada N, Konno A, Yamashita T, *et al.* Serum level of soluble ICAM-1 in subjects with nasal allergy and ICAM-1 mRNA expression in nasal mucosa. *Arerugi* 1993; **42:** 87–93.
33. Himi T, Kamimura M, Kataura A, Imai K. Quantitative analysis of soluble cell adhesion molecules in otitis media with effusion. *Acta Otolaryngol (Stockhol)* 1994; **114:** 285–288.
34. Smith CW, Rothlein R, Hughes BJ, Mariscalco MM, Rudloff HE, Schmalstieg FC, Anderson DC. Recognition of an endothelial determinant for CD18-dependent human neutrophil adherence and transendothelial migration. *J Clin Invest* 1988; **82:** 1746–1756.
35. Jung TTK. Prostaglandins, leukotrienes, and other arachidonic metabolites in the pathogenesis of otitis media. *Laryngoscope* 1988; **98:** 980–993.
36. Cauwenberge PB, Bernstein JM. Inflammatory mediators in middle ear disease. In: Berstein JM, Ogra PL, eds. *Immunology of the ear.* New York: Raven Press, 1987; 331–343.
37. Ganbo T, Hisamatsu K, Shimomura S, Nakajima T, Inoue H, Murakami Y. Inhibition of mucociliary clearance of the eustachian tube by leukotriene C4 and D4. *Ann Otol Rhinol Laryngol* 1995; **104:** 231–236.
38. Ganbo T, Hisamatsu K, Kikushima K, Nakajima M, Inoue H, Murakami Y. Effects of platelet activating factor (PAF) on mucociliary clearance of the eustachian tube. *Ann Otol Rhinol Laryngol* (in press).

**Received 2 June 1995; accepted 19 June 1995**
